# Selective accumulation of langerhans-type dendritic cells in small airways of patients with COPD

**DOI:** 10.1186/1465-9921-11-35

**Published:** 2010-03-22

**Authors:** Geert R Van Pottelberge, Ken R Bracke, Ingel K Demedts, Kim De Rijck, Susanne M Reinartz, Cornelis M van Drunen, Geert M Verleden, Frank E Vermassen, Guy F Joos, Guy G Brusselle

**Affiliations:** 1 Laboratory for Translational Research in Obstructive Pulmonary Diseases, Department of Respiratory Medicine, Ghent University Hospital, Ghent, Belgium; 2 Department of Otorhinolaryngology, Academic Medical Center, Amsterdam, Netherlands; 3 Department of Respiratory Medicine, University Hospital Gasthuisberg, Catholic University of Leuven, Belgium; 4 Department of Thoracic and Vascular Surgery, Ghent University Hospital, Ghent, Belgium

## Abstract

**Background:**

Dendritic cells (DC) linking innate and adaptive immune responses are present in human lungs, but the characterization of different subsets and their role in COPD pathogenesis remain to be elucidated. The aim of this study is to characterize and quantify pulmonary myeloid DC subsets in small airways of current and ex-smokers with or without COPD.

**Methods:**

Myeloid DC were characterized using flowcytometry on single cell suspensions of digested human lung tissue. Immunohistochemical staining for langerin, BDCA-1, CD1a and DC-SIGN was performed on surgical resection specimens from 85 patients. Expression of factors inducing Langerhans-type DC (LDC) differentiation was evaluated by RT-PCR on total lung RNA.

**Results:**

Two segregated subsets of tissue resident pulmonary myeloid DC were identified in single cell suspensions by flowcytometry: the langerin+ LDC and the DC-SIGN+ interstitial-type DC (intDC). LDC partially expressed the markers CD1a and BDCA-1, which are also present on their known blood precursors. In contrast, intDC did not express langerin, CD1a or BDCA-1, but were more closely related to monocytes.

Quantification of DC in the small airways by immunohistochemistry revealed a higher number of LDC in current smokers without COPD and in COPD patients compared to never smokers and ex-smokers without COPD. Importantly, there was no difference in the number of LDC between current and ex-smoking COPD patients.

In contrast, the number of intDC did not differ between study groups. Interestingly, the number of BDCA-1+ DC was significantly lower in COPD patients compared to never smokers and further decreased with the severity of the disease. In addition, the accumulation of LDC in the small airways significantly correlated with the expression of the LDC inducing differentiation factor activin-A.

**Conclusions:**

Myeloid DC differentiation is altered in small airways of current smokers and COPD patients resulting in a selective accumulation of the LDC subset which correlates with the pulmonary expression of the LDC-inducing differentiation factor activin-A. This study identified the LDC subset as an interesting focus for future research in COPD pathogenesis.

## Background

Chronic Obstructive Pulmonary Disease (COPD) is characterized by destruction of alveolar walls (emphysema) and obstructive bronchiolitis resulting in a progressive airflow limitation that is not fully reversible [1]. In industrialized countries, cigarette smoke is the most frequently encountered risk factor for the development of COPD. Currently, COPD is the fourth leading cause of death worldwide and according to the World Health Organization (WHO), mortality will further increase in the next 20 years [2,3]. The exact pathogenetic mechanisms of continuing destructive inflammation in this disease are not completely understood. Several studies identified the important role of the activated innate immune response in the pathogenesis of COPD, with neutrophils and macrophages as major effector cells, inducing tissue destruction by proteolysis and oxidative stress [4-6]. Other studies addressed the role of the adaptive immune response in COPD, with increased numbers of lymphoid follicles and the presence of cytotoxic CD8+ T cells and B cells, reflecting a sustained immune response, even after smoking cessation [7-10].

Dendritic cells (DC) form the crucial link between the innate and adaptive immunity. Immature DC form a network in the different layers of the airway mucosa, specialized in internalizing antigens. Upon recognition of antigen in the context of a pathogen- or damage- associated danger signal, DC undergo a maturation process and migrate towards the draining lymph nodes. Mature DC present the processed antigen on Major Histocompability Complex (MHC) molecules and interact with naïve T lymphocytes to form an immunological synapse, selecting T cells that will target the presented antigen with specialized effector functions. In this key position, DC are able to orchestrate the nature and magnitude of the adaptive immune response to different antigens [11,12].

Several groups already identified different subsets of DC in the human lung, mainly using antibodies against epitopes also present on circulating blood DC (such as Blood Dendritic Cell Antigen (BDCA) 1-4 and CD11c) [13-16]. However, no flowcytometric data are available about pulmonary tissue-resident myeloid DC, such as Langerhans-type DC (LDC) and Interstitial-type DC (intDC), two major DC subsets that are well characterized in the skin. In general, LDC are identified by the C-type lectin langerin (CD 207) and the presence of Birbeck granules. They are mainly localized in the epithelium, and are involved in activating cellular/cytotoxic immune responses. IntDC are identified by the C-type lectin DC-SIGN (Dendritic Cell-Specific Intracellular adhesion molecule-3-grabbing Non-integrin, CD 209), are localized in the subepithelial layers and are known to activate humoral immune responses [17]. CD34+ stem cells and monocytes are known precursors of LDC and intDC [18,19]. In addition, in vitro studies have shown that BDCA-1+ CD1a+ blood DCs are the direct precursors of LDC [20].

Evidence from experimental mouse models suggests a role for DC in the pathogenesis of COPD [21]. Recently, we showed that LDC accumulate in the small airways of patients with COPD [22], while others reported a decreased number of electronmicroscopic defined DC in bronchial biopsies of smoking COPD patients [23]. In contrast, Verhoeven et al and a recent study by Tsoumakidou et al found no differences in CD1a positive DCs in bronchial biopsies and small airways of COPD patients compared to asymptomatic smokers [24,25]. Importantly, several studies indicated that smoking as such could alter the numbers of DCs in the lung [26-28].

In this study, we aimed to identify and characterize tissue resident pulmonary LDC and intDC, investigating the interrelationship of different myeloid DC markers such as langerin, BDCA-1, BDCA-3, CD1a and DC-SIGN. In addition, we aimed to quantify immature myeloid DC in small airways of never smokers and smokers with or without COPD, taking into account whether they were current or ex- smokers. Finally, we examined the pulmonary expression of factors such as activin-A [29], Notch ligand Delta-1 [30], RANK-ligand [31] and interleukin 15 [32], known from in vitro studies to modulate the differentiation process of monocytes towards myeloid DC.

This study shows the presence of two segregated myeloid tissue resident pulmonary DC populations: the intDC and the LDC. In the small airways of current smokers without COPD and in COPD patients, there is a selective accumulation of the LDC subset correlating with the pulmonary expression of the LDC inducing differentiation factor activin-A. This evidence is compatible with an altered differentiation process of myeloid DC in the small airways and identifies the LDC as an important focus for future research in COPD pathogenesis.

## Methods

### Lung tissue

Lung tissue was obtained from surgical lung resection specimens of patients diagnosed with solitary pulmonary tumours at the Ghent University Hospital. Lung tissue at maximum distance from the pulmonary lesion and without signs of retro-obstructive pneumonia or tumour invasion was collected by a pathologist. None of the patients operated for malignancy were treated with neo-adjuvant chemotherapy. Lung tissue from end-stage COPD was obtained from explant lungs from patients undergoing lung transplantation (University Hospital Gasthuisberg, Leuven, Belgium). The study population from which tissue was obtained partially overlaps with and extends the one described in our previous study [22]. All patients signed informed consent prior to surgery and were interviewed about their smoking habits and medication use. Patients were classified as ex-smokers when they had quit smoking at least 1 year prior to surgery. COPD diagnosis and severity was defined using pre-operative spirometry according to the GOLD classification [1]. This study was approved by the Medical Ethical Committees of the Ghent University Hospital and the University Hospital Gasthuisberg Leuven.

Between the year 2002 and 2008, small tissue blocks from the peripheral lung tissue of 85 patients were stored for immunohistochemical analysis. Samples were immediately placed in OCT (Tissue-Tek, Sakura Finetek, Zoeterwoude, the Netherlands), snap-frozen in liquid nitrogen cooled isopentane and stored at minus 80° Celsius. Other samples from the same specimen were fixed in paraformaldehyde 4% (Sigma, Bornem, Belgium) during 12 hours and embedded in paraffin wax. In 44 of the 85 patients, RNA was extracted from a part of the resection specimen which was stored in RNA stabilizing agent (RNAlater, Qiagen, Hilden, Germany) at minus 80° Celcius. At the moment of designing the PCR experiments in total lung RNA, a set of 11 RNA samples from new subjects (not present in the immunohistochemical study) was additionally included.

### Flowcytometry

In a separate study, the lung tissue sample from 6 individual cases (included in 2008-2009) was processed to obtain single cell suspensions for flowcytometry as described previously [13]. There was no overlap between the subjects in the flowcytometric study and the immunohistochemical study. Single cells were pre-incubated with human IgG to reduce non-specific binding. Monoclonal antibodies used are FITC conjugated anti-human CD3 (clone UCTH1) and CD19 (clone HIB19), PERCP conjugated anti-HLA-DR (clone L243), APC conjugated anti- human CD14 (clone M5E2) and PE or APC conjugated anti human DC-SIGN (clone DCN46) (all from BD Biosciences, Erembodegem, Belgium). PE conjugated anti-human langerin (CD207, clone DCGM4) was purchased at Immunotech-Beckman-Coulter, Marseille, France. Anti-human biotinylated CD1a (clone HI149) was purchased at eBioscience, San Diego, CA. APC conjugated anti human BDCA-1 (CD1c, clone AD5-8E7), BDCA-2 (CD303, clone AC141), BDCA-3 (CD141, clone AD5-14H12) and BDCA-4 (CD304, clone AD5-17F6) were purchased at Miltenyi Biotec, Bergisch Gladbach, Germany. APC conjugated anti-human CD163 was purchased at R&D systems, Abingdon, UK. PERCP conjugated streptavidin was purchased at BD Biosciences, Erembodegem, Belgium. Appropriate monoclonal antibodies for isotype control staining were used.

Flowcytometric data acquisition was performed on a FACS Calibur equipped with 488 and 633 nm lasers and running Cellquest Software (Becton Dickinson, San Diego, CA, USA). Flowjo software was used for data analysis (Treestar, OR, USA).

### Histology

7 μm thick cryosections were cut on poly-L-lysine-coated microscopic slides (Sigma, Bornem, Belgium). Sections were dried for 24 hours and stored at minus 80° Celsius until use. Prior to the immunohistochemical procedure, cryosections were defrosted to room temperature, dried and fixed in aceton for 10 minutes. After fixation, tissue sections were rinsed with phosphate-buffered saline (PBS, pH 7.8).

Sections were incubated with mouse anti-human monoclonal antibody directed against BDCA-1 (AD5-8E7) (Miltenyi Biotec, Bergisch Gladbach, Germany) following incubation with normal goat serum (CLB, Amsterdam, the Netherlands). Sections were then incubated with biotinylated goat anti-mouse antibody (Biogenics, Klinipath, Duiven, the Netherlands). Next, sections were incubated with streptavidin alkaline phosphatase (ss-AP, Biogenics, Klinipath, Duiven, the Netherlands). Sections were then rinsed with PBS containing TRIS buffer (0.2 mol/L, pH 8.5) and incubated with new fuchsine (Chroma, Kongen, Germany) substrate (containing levamisole to block endogenous alkaline phosphatase enzyme activity), counterstained with hematoxylin and mounted in Vecta Mount (Vector, Burlingame, CA, USA).

Aceton-fixed cryosections were stained with mouse anti-human DC-SIGN ((clone DCN46), BD Biosciences, Erembodegem, Belgium) following incubation with blocking reagent (Roche 1096176, Basel, Switzerland). Sections were then incubated with poly-alkaline phosphatase goat anti-mouse (Klinipath, Duiven, the Netherlands). Sections were incubated with new fuchsine (Dako, Heverlee, Belgium) with levamisole during 7 minutes, counterstained with Mayer's hematoxylin (Sigma, Belgium), rinsed with distilled water and mounted in Aquatex (Klinipath).

Immunohistochemical staining for langerin was carried out on cryosections as described previously [22].

For immunohistochemical double staining, aceton fixed cryosections were incubated with blocking reagent (Ultra V Block : Klinipath: TA-125-UB), followed by the first monoclonal antibody (anti human BDCA-1, (clone AD5-8E7) (Miltenyi Biotec, Bergisch Gladbach, Germany) or anti human DC-SIGN (clone DCN46, BD Biosciences, Erembodegem, Belgium) during 1 hour at room temperature. Sections were then incubated with poly-Alkaline phosphatase (DPVM 55AP, Klinipath, Duiven, The Netherlands) stained with new fuchsin as described above. After rinsing, streptavidin/biotin blocking was applied (SP-2002, Vector, Burlingame, CA) followed by blocking reagent (Roche 1096176, Basel, Switzerland). Sections were incubated overnight with mouse anti-human CD207 (langerin, clone DCGM4, Immunotech, Marseille, France) diluted in PBS. Sections were rinsed with PBS and incubated with biotinylated goat-anti-mouse IgG1 (Southern Biotech Birmingham, USA). Sections were rinsed in PBS and incubated with streptavidin-AP (Vector, Burlingame, CA). Finally, sections were stained with Vector Blue diluted in PBS with 0.3% Triton (Sigma, Belgium) at pH 8.2 combined with levamisole. After terminating the staining process with PBS with 0.3% Triton at pH 7.5, sections were rinsed with distilled water and covered with Aquatex as described above.

3 μm thick paraffin embedded sections were cut on poly-L-lysin coated slides. After dewaxing with Ultra Clear (Klinipath, Duiven, The Netherlands) and rehydration, antigen retrieval was performed using preheated Citrate buffer pH 6.0 (ScyTek Laboratories, Logan, Utah, USA) 10% at 78°C. After blocking of endogenous peroxidase activity with 3% hydrogen peroxide (Dako, Heverlee, Belgium) and application of blocking reagent (Roche 1096176, Basel, Switzerland) 1% in PBS with 0.3% Triton, sections were incubated with mouse anti-human CD1a monoclonal antibody (M3571, Dako, Heverlee, Belgium) followed by incubation with biotinylated link antibody and application of streptavidin-HRP (LSAB system K0679, DAKO, Heverlee, Belgium). Slides were rinsed in PBS containing 0.3% Triton. Finally, diaminobenzidine substrate was added for 30 minutes, sections were rinsed in demineralised water, counterstained with Mayer's hematoxylin (Sigma, Belgium), dehydrated and mounted in DPX (Klinipath, Duiven, The Netherlands).

Immunohistochemical staining for for Activin-A was also performed on paraffin-embedded sections, using the same dewaxing and rehydration protocol as described above. Antigen retrieval was performed using EDTA-buffer. After blocking for endogenous peroxidase activity and application of Fc block, slides were incubated with anti-human Activin-A ((Clone E4) ABD Serotec, Kidlington, United Kingdom) during 12 h at 4°C. Next, sections were further stained as described in the protocol for CD1a.

For each primary antibody used, appropriate isotype control stainings were performed.

### Image analysis

Images of tissue sections were recorded using a computerized image analysis system (KS400, Zeiss, Oberkochen, Germany). Airways without cartilage that had a perimeter of the basement membrane of less than 6000 μm were selected for analysis [7]. The area of epithelium was defined by the region between the luminal border and the basement membrane, the lamina propria as the region between the basement membrane and the outer edge of the smooth muscle and the adventitia as the region between the outer border of the smooth muscle and the outer border of the small airway. The total airway wall was defined as the sum of these 3 regions. The number of positive cells in the epithelium was counted and the results were normalized to the area of the epithelium and to the length of the basement membrane. For the lamina propria, adventitia and total airway wall, the number of positive cells were normalized to the surface of the respective area. Cells were regarded positive when showing DC morphology and contained a nucleus. The observer (GRVP) was blinded for clinical data. A random sample of 45 slides was analysed by a second blinded observer (KD), showing good interobserver agreement (paired T test p = 0.18) and a good correlation between the two observers (p < 0.001, Pearson correlation coefficient 0.85).

### mRNA expression study

Total lung RNA was extracted with the RNeasy Mini Kit (Qiagen, Hilden, Germany). RNA quality was checked on a bioanalyser and samples with an RNA-integrity number (RIN) below 5.5 were excluded from the analysis. Subsequently cDNA was obtained by reverse transcription of RNA with the Transcriptor First Strand cDNA synthesis kit (Roche, Basel, Switzerland) following manufacturer's instructions and using a 2:1 ratio of hexa:oligodT primers. Expression of target genes Activin A (Inhibin beta A), RANKL, Notch Ligand Delta-1 and IL-15 and reference genes GAPDH (glyceraldehyde-3-phosphate dehydrogenase), HPRT1 (hypoxanthine phosphoribosyltransferase 1) and PPIA (peptidylprolyl isomerase 1) mRNA was analysed with the TaqMan Gene Expression Assays (Applied Biosystems, Forster City, CA, USA). Real-time PCR reactions were performed in duplicate using diluted cDNA template and the LightCycler480 Probes Master (Roche, Basel, Switzerland). A standard curve derived from the serial dilutions of a mixture of all samples was included on each plate. Amplifications were performed on a LightCycler480 detection system (Roche, Basel Switzerland) with the following cycling conditions: 10 min incubation at 95°C and 50 cycles of 95°C for 10 sec and 60°C for 15 sec. Data were processed using the standard curve based method. Expression of target genes was corrected by a normalisation factor that was calculated based on the expression of three reference genes (GAPDH, HPRT1, PPIA), using the geNorm applet according to the guidelines and theoretical framework previously described http://medgen.ugent.be/~jvdesomp/genorm/[33] In 44 patients, both total lung RNA and langerin stained cryosections were available.

### Statistical analysis

Statistical analysis was carried out in SPSS 16.0 (SPSS inc. Chicago, IL, USA). When evaluating differences in continuous variables between multiple independent groups, the Kruskal-Wallis test was used. Where values of probability were <0.05, selected pairs of groups were investigated by the Mann-Whitney U test. Correlation coefficients were calculated using Spearman's rank method. Linear regression analysis was performed on log-transformed data, using the enter method. P values < 0.05 were considered significant.

## Results

### Characterization of pulmonary Langerhans-type and interstitial-type DC

**Figure 1:**
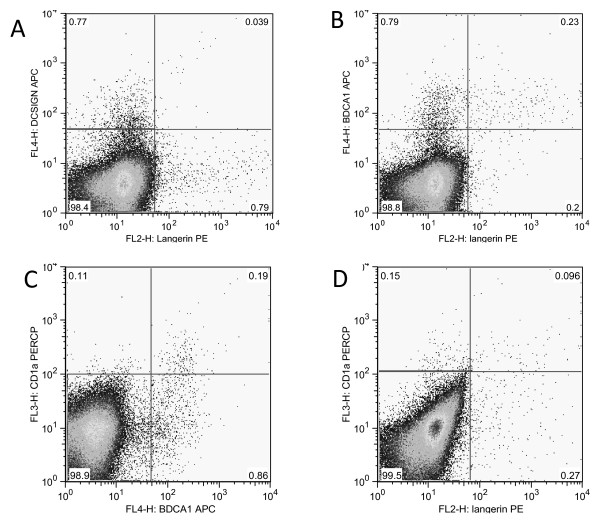
**Flowcytometric identification of langerin+ Langerhans type dendritic cells and DC-SIGN + interstitial type dendritic cells on single cell suspensions of digested human lung tissue, using the low autofluorescent, CD3 and CD19 negative gating strategy**. (A): Langerin+ and DC-SIGN+ dendritic cells are two separate dendritic cell populations. (B-D): interrelationship of langerin, CD1a and BDCA-1 on low autofluorescent, CD3 and CD19 negative dendritic cells. (representative of 6 independent experiments).

Figure 1 shows the flowcytometric identification of langerin (CD207)+ LDC and DC-SIGN (CD209)+ intDC in single cell suspensions of digested human lung tissue, using the previously described low autofluorescent, CD3 negative, CD19 negative gating strategy [13]. The clinical characteristics of the study population and the proportion of each DC marker within the HLA-DR positive population are shown in table 1.

**Table 1:** Characteristics of the Study Population (flowcytometry) (n = 6)

subject	1	2	3	4	5	6
**gender**	M	M	F	M	M	M
**Age (years)**	60	76	63	67	77	69
**Smoking history (packyear)**	0	0	40	40	35	50
**Current or ex smoker**	NA	NA	current	current	ex	ex
**FEV1 (%pred)**	77.0	111.0	94.0	97.0	74	75.0
**FEV**_**1**_**(L)**	2.4	3.0	1.8	2.9	1.9	1.9
**FEV**_**1**_**/FVC (%)**	70	76	68	68	64	58
**LABA (Yes/No)**	No	No	No	No	No	No
**Inhaled CS (Yes/No)**	No	No	No	No	No	No
**Systemic CS (Yes/No)**	No	No	No	No	No	No
**Study group**	Never Smoker	Never Smoker	COPD*	COPD*	COPD*	COPD*

**% Langerin + cells**	2.0	5.4	1.7	1.0	0.9	16.5
**% DC-SIGN + cells**	2.0	14.1	0.8	0.7	1.0	2.9
**% BDCA-1 + cells**	4.2	14.2	2.5	2.0	2.5	13.4
**% BDCA-3 high + cells**	20.0	3.7	6.0	7.9	2.5	4.0
**% BDCA-1+ cells on LDC**	73.5	88.0	80.8	75.0	64.1	14.2

Data are provided per subject. CS: corticosteroids; FEV1: forced expiratory volume in one second postbronchodilator; FVC: forced vital capacity; LABA : long acting β 2 agonist; M: Male, F: Female; (*): spirometry-based diagnosis of COPD according to GOLD criteria.

Flowcytometric data on single cell suspensions are presented as % of HLA-DR positive cells within the low autofluorescent, CD3 negative, CD19 negative cell population. LDC: langerhans-type dendritic cell

LDC and intDC were consistently identified as two separate populations (figure 1A). A large proportion of LDC expressed the myeloid DC marker BDCA-1 (figure 1B), but in multiple independent experiments, a small population of BDCA-1 negative LDC was consistently found. The majority of CD1a+ DC expressed BDCA-1 (figure 1C), but only a minor subset of CD1a+ DC expressed langerin. Moreover, the majority of LDC was CD1a negative (figure 1D).

**Figure 2:**
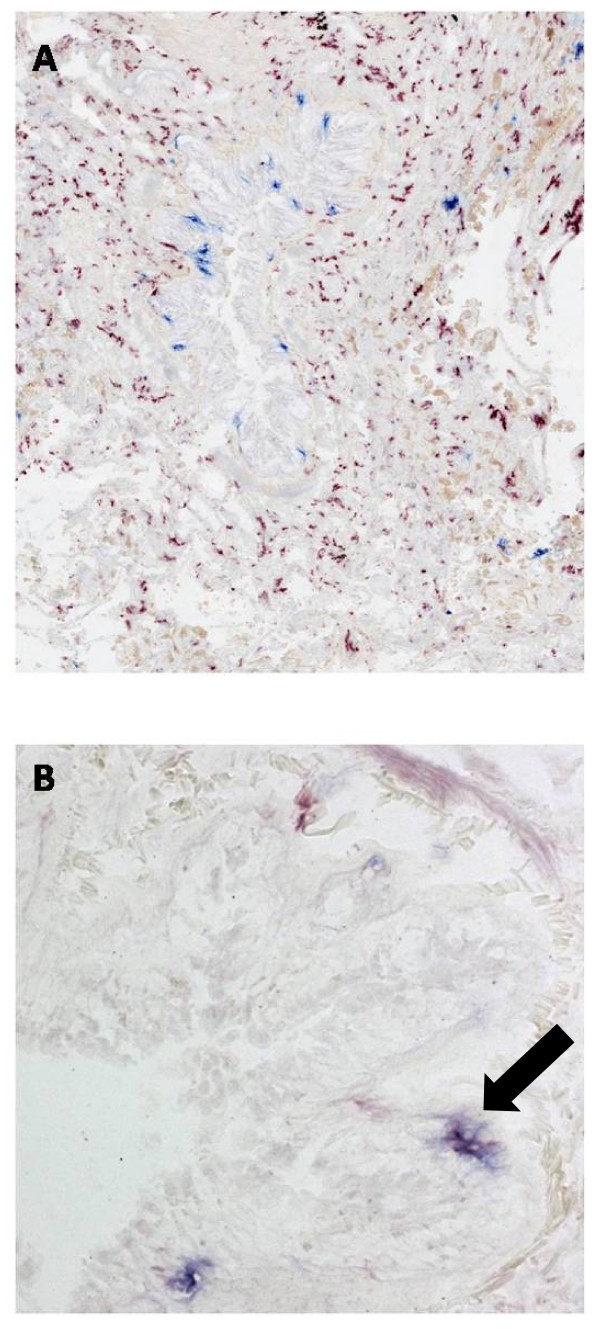
**Immunohistochemical double staining for myeloid dendritic cell markers.** (A): Immunohistochemical double staining for DC-SIGN+ interstitial type dendritic cells (red) and langerin+ Langerhans type dendritic cells (Blue) in small airway of human lung. Langerin+ cells are mainly present in the epithelium, whereas DC-SIGN+ cells are localized in the lamina propria and adventitia. There are no double positive cells, confirming the segregation of these two cell types in human lung. (B): Immunohistochemical double staining for BDCA-1+ myeloid dendritic cells and langerin+ Langerhans type dendritic cells in small airway of human lung. A single positive BDCA-1 + cell (red), a langerin positive cell (blue) and a double positive cell (purple, marked with an arrow) are present.

Immunohistochemical double staining on cryosections of lung resection specimens confirmed the segregation of LDC and intDC, the former mainly present in the epithelium, the latter in the lamina propria and the adventitia (figure 2A). Double staining for langerin and BDCA-1 confirms the presence of a double positive and the respective single positive populations (figure 2B).

**Figure 3:**
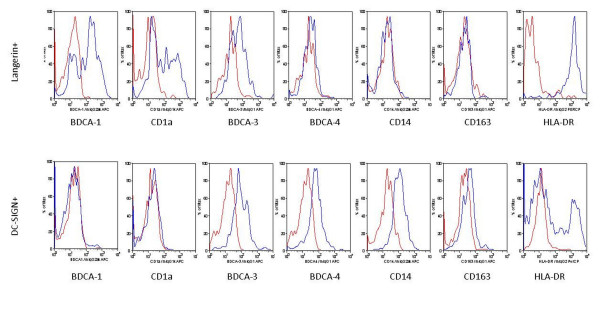
**Characterization of langerin positive and DC-SIGN positive dendritic cels**. Expression of BDCA-1, CD1a, BDCA-3, BDCA-4, CD14, CD163 and HLA-DR are represented by the blue histograms. Red histograms represent the matched isotype control staining. Results are representative of 6 independent experiments.

IntDC showed a completely different expression profile compared to LDCs. Representative histograms comparing the expression profile on LDC and intDC are shown in figure 3A and 3B. In addition, quantitative data on these expression profiles are shown in Table 2. IntDC did not express BDCA-1 and CD1a. The expression of BDCA-4 tended to be higher in intDC than in LDC. Another blood dendritic cell antigen (BDCA-3) was expressed on both subsets. IntDC expressed CD14 and a variable degree of CD163, showing their close relationship with monocytes and macrophages. Nearly all LDC express HLA-DR (97%), whereas only a proportion (40%) of intDCs expressed this marker.

**Table 2:** Expression of Surface Markers on interstitial-type and langerhans-type DC (flowcytometric analysis of lung digest)

Surface marker	% positive cells			Mean Fluorescence Intensity		
	intDC	LDC	p	intDC	LDC	p
**BDCA-1**	6.3 [1.7-9.6]	72.6 [42.1-81.7]	**	9.6 [3.1-16.5]	60.2 [48.3-140.0]	**
**CD1a**	3.1 [0.3-5.2]	54.1 [23.0-73.8]	*	13.4 [4.4-30.3]	112.4 [38.2-203.0]	*
**BDCA-3**	54.3 [13.5-98.1]	56.2 [3.2-97.3]	ns	54.0 [44.3-168.0]	35.4 [21.2-156.0]	ns
**BDCA-4**	15.0 [1.9-57.1]	4.6 [0.0-15.0]	ns	33.0 [8.6-89.6]	14.5 [8.6-38.5]	ns
**CD163**	4.5 [3.0-46.2]	4.3 [0.6-26.8]	ns	27.1 [11.9-186.0]	20.0 [3.7-127.0]	ns
**CD14**	57.2 [20.4-81.5]	10.2 [2.5-23.1]	*	56.3 [11.5-207.0]	12.6 [3.6-87.8]	ns
**HLA-DR**	56.3 [21.4-98.5]	88.7 [73.1-98.7]	ns	132.0 [31.7-2399]	544.0 [484.0-1629.0]	ns

Data are expressed as median [range]

intDC: interstitial-type dendritic cell; LDC: langerhans-type dendritic cell

p value compairing two groups (Mann-Whitney-U test): *: p < 0.05; **: p < 0.01; ns: not significant

**Figure 4:**
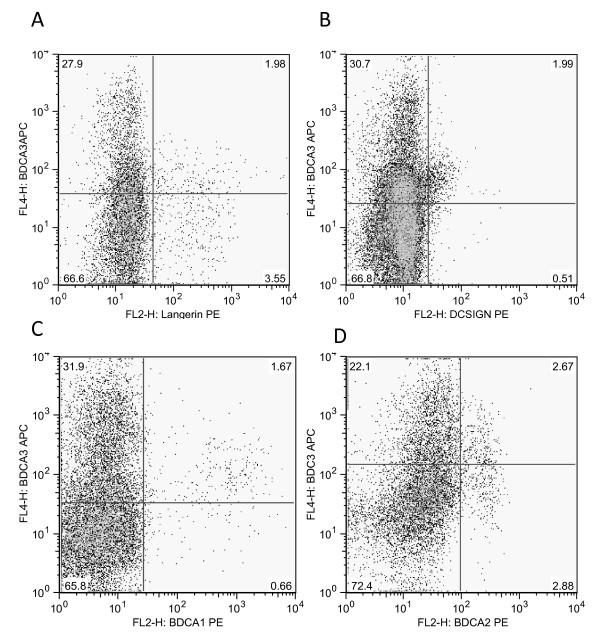
**Interrelationship between BDCA-3 expression and (A) langerin, (B) DC-SIGN, (C) BDCA-1 and (D) BDCA-2 on low autofluorescent, CD3 negative, CD19 negative, HLA-DR positive cells**.

Both intDC and LDC expressed BDCA-3 on flowcytometric analysis, which is the previously described marker for the pulmonary myeloid DC type 2 subset [13]. However, as shown in figure 3, LDC and intDC both expressed BDCA-3 at an intermediate level. We consistently found in all experiments a low autofluorescent, CD3 negative, CD19 negative, HLA-DR positive population that expressed BDCA-3 at a high level (Figure 4) This BDCA-3 high cell population did not express BDCA-1, DC-SIGN, langerin or BDCA-2 (a pDC marker), suggesting that this is also a separate myeloid DC population.

### Distribution and quantification of dendritic cells in small airways

#### Clinical characteristics of the study population

Table 3 shows the clinical characteristics of the study population consisting of 85 patients (11 never-smokers, 14 current smokers without COPD, 14 ex-smokers without COPD and 46 COPD patients).

**Table 3:** Characteristics of the Study Population (immunohistochemical study) (n = 85)

	Never Smokers	Current Smokers without COPD	Ex-Smokers without COPD	COPD I-II Current Smokers	COPD I-II Ex-Smokers	COPD III-IV Ex-Smokers
**n**	11	14	14	23	13	10
**Sex ratio (M/F)**	3/8	9/5	12/2	22/1	13/0	4/6
**Age (years)**	57.6 (11.5)	55.6 (11.0)	63.7 (7.4)^†^	63.0 (9.0)^†^	69.5 (6.9)*^†‡Δ^	57.3 (5.0)^‡Δ∞^
**Smoking history (packyear)**	N.A.	31.4 (12.0)	39.1 (35.6)	46.5 (21.1)^†^	42.5 (23.2)	44.5 (41.8)
**Years quit smoking**	N.A.	0.2 (0.2)	18.0 (10.3)^†^	0.1 (0.2)^†‡^	9.9 (7.3)^†‡Δ^	5.1 (2.5)^†‡Δ^
**FEV**_**1**_**(%pred)**	102.4 (13.0)	102.3 (13.7)	102.6 (19.1)	74.2 (13.2)*^†‡^	83.6 (11.0)*^†‡^	27.0 (9.9)*^†‡Δ∞^
**FEV**_**1**_**(L)**	2.8 (0.9)	3.1 (0.4)	3.2 (1.0)	2.4 (0.5)^†‡^	2.5 (0.4)^†^	0.7 (0.3)*^†‡Δ∞^
**FEV**_**1**_**/FVC (%)**	77.0 (7.1)	77.9 (4.7)	76.2 (5.8)	57.3 (8.0)*^†‡^	60.3 (5.3)*^†‡^	37.7 (10.9)*^†‡Δ∞^
**LABA (Y/N)**	0/11	0/14	0/14	6/17	3/10	10/0
**Inhaled corticosteroids (Y/N)**	0/11	0/14	0/14	5/18	2/11	10/0
**Inhaled corticosteroid dose (μg BDP/24 h)**	0	0	0	369.6 (771.9)	307.7 (751.1)	1660 (550.2)*^†‡Δ∞^
**Systemic corticosteroids (Y/N)**	0/11	0/14	1/13	1/22	0/13	7/3
**Systemic corticosteroid dose (mg prednisolon/24 h)**	0	0	0.4 (1.6)	0.4 (2.1)	0	5.5 (4.4)*^†‡Δ∞^

Data are expressed as mean (standard deviation);

* p < 0.05 vs never smoker; † p < 0.05 versus current smoker without COPD; ‡ p < 0.05 versus ex-smoker without COPD; Δ p < 0.05 versus COPDI-II current smoker; ∞ p < 0.05 versus COPD I-II ex-smoker.

FEV1: forced expiratory volume in one second;

FVC: forced vital capacity;

LABA : long acting β 2 agonist; BDP: beclomethasone diproprionate.

#### DC-SIGN positive interstitial-type DC

**Figure 5:**
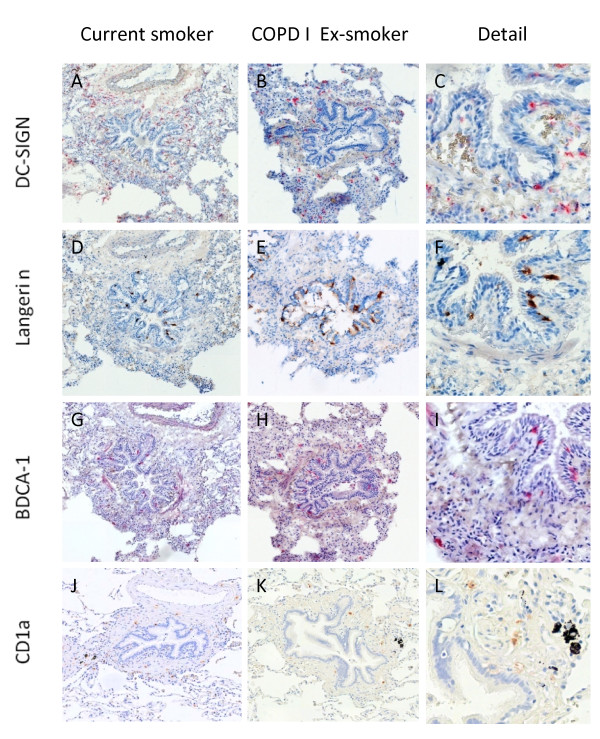
**Immunohistochemical staining for dendritic cell markers DC-SIGN (CD209) (A), (B), (C); langerin (CD207) (D), (E), (F); BDCA-1 (CD1c) (G), (H), (I); and CD1a (J), (K), (L) in sections of a small airway of a current smoker without COPD and an ex-smoking COPD patient GOLD stage I**. Original magnification: 100×. Pictures in the right column show a detail:(C), (F) and (I) are detailed pictures from the first column. (L) is a detail from the second column (K).

**Figure 6:**
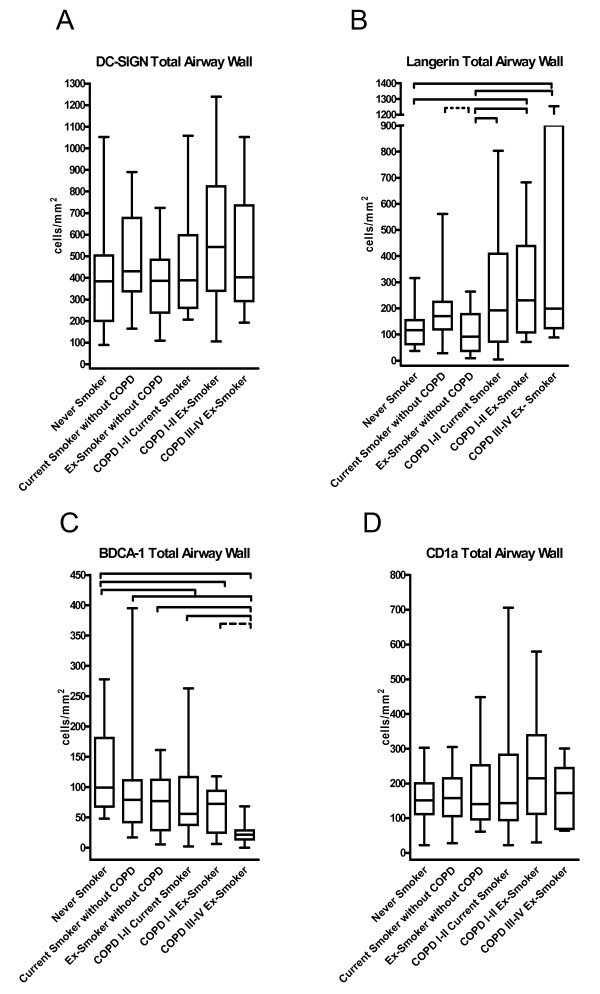
**Box and whisker plots: Quantification of DC-SIGN positive (interstitial-type) dendritic cells (A), Langerin positive (Langerhans-type) dendritic cells (B), BDCA-1 positive myeloid dendritic cells (C) and CD1a positive dendritic cells (D) in small airways of never smokers, current smokers without COPD, ex-smokers without COPD, current smoking COPD patients GOLD I-II, ex-smoking COPD patients GOLD I-II and ex-smoking COPD patients GOLD stage III&IV**. Data are presented as number of dendritic cells/area of total airway wall (cells/mm^2^). Significant differences between 2 groups (p values <0.05), generated by the Mann-Whitney-U test after analysis of variance are marked with a full line, p values between 0.05 and 0.08 are marked by a dashed line.

**Figure 7:**
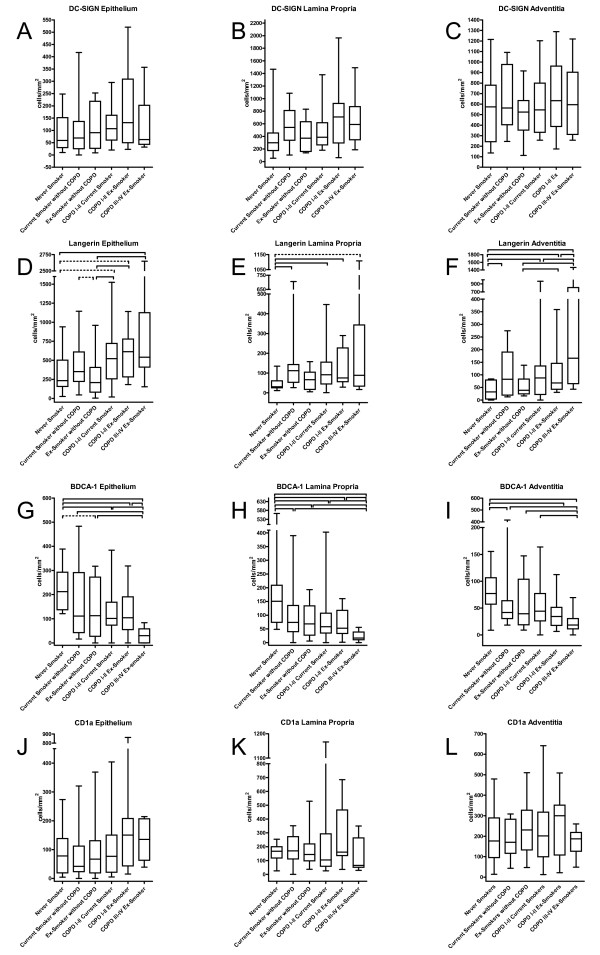
**Box-and Whisker plots: Quantification of myeloid dendritic cells in small airways of never-smokers (n = 11), current smokers without COPD (n = 14), ex-smokers without COPD (n = 14), current smoking COPD patients GOLD stage I&II (n = 23), ex-smoking COPD patients GOLD stage I&II (n = 13) and ex-smoking COPD patients GOLD stage III&IV (n = 10)**. A-C: DC-SIGN positive intersititial type dendritic cells. D-F: langerin positive Langerhans-type dendritic cells, G-I: BDCA-1 positive dendritic cells and J-L: CD1a positive dendritic cells. Data are presented as number of dendritic cells/area of epithelium, lamina propria or adventitia (cells/mm^2^). Significant differences between two groups (p values < 0.05), generated by Mann-Whitney-U test after analysis of variance are marked with a full line, p values between 0.05 and 0.08 are marked with a dashed line.

Figure 5A-C shows representative cryosections of DC-SIGN positive DC in small airways. These cells were mainly located in the lamina propria and adventitia. There were no significant differences in the number of DC-SIGN positive DC between the study groups in the total airway wall (figure 6A), the epithelium, lamina propria and adventitia (figure 7:A-C). No significant correlation with FEV1 was found (data not shown).

#### Langerin positive Langerhans-type DC

Representative cryosections showing langerin+ DC are displayed in figure 5 D-F. Langerin+ DCs were mainly found in the epithelium and to a lesser extent in the lamina propria and the adventitia. In the total airway wall, the number of langerin+ DC was significantly higher in all COPD groups compared to ex-smokers without COPD (figure 6B). This increase was due to a significant accumulation of these cells in the epithelium and adventitia, with additional higher numbers of langerin+ DC in the adventitia of the patients with COPD GOLD stage III&IV (p = 0.045). (figure 7D-F).

In current smokers without COPD, the number of langerin+ DCs tended to be higher in the total airway wall (p = 0.060) and in the epithelium (p = 0.062) compared to ex-smokers without COPD. When the number of cells in the epithelium was expressed per unit of length of the basement membrane, significantly higher numbers were observed in the epithelium of current smokers compared to ex-smokers without COPD (p = 0.012) (data not shown). In addition, the number of langerin+ DC was significantly higher in the lamina propria and adventitia of current smokers without COPD compared to never smokers (p = 0.008 and p = 0.033). There were no significant differences between never smokers and ex-smokers without COPD in any layer of the small airway. Importantly, there were also no differences between current and ex-smoking COPD GOLD stage I/II. There were significant negative correlations between the forced expiratory volume in 1 second (FEV1) % predicted and the number of langerin positive cells in the epithelium (correlation coefficient r_s _-0.39; p < 0.001), lamina propria (r_s _-0.30, p = 0.006), adventitia (r_s _-0.38; p < 0.001) and total airway wall (r_s _-0.36; p = 0.001). This association between FEV_1 _(% predicted) and the number of langerin+ DC in the small airways remained significant, even after adjustment for possible confounders (age, gender, amount of packyears smoked, current versus non-current smoker, treatment with inhaled corticosteroids and treatment with oral corticosteroids) by linear regression analysis (Table 4).

**Table 4:** Linear Regression Analysis (Quantification Langerin+ DC in small airways)

DC surface marker	**R**^**2**^	Predictor Variable	β	p
Langerin+ DC in Total Airway Wall	0.23	FEV_1_(% pred)	-0.436	**0.002**
		Age	-0.088	0.441
		Gender	0.186	0.105
		Packyear	0.065	0.592
		Smoking status	0.011	0.920
		Inhaled CS	0.006	0.968
		Oral CS	0.047	0.705

Langerin+ DC in Epithelium	0.20	FEV_1_(% pred)	-0.398	**0.006**
		Age	-0.049	0.674
		Gender	0.130	0.264
		Packyear	0.031	0.805
		Smoking status	0.090	0.427
		Inhaled CS	0.019	0.898
		Oral CS	0.046	0.715

Langerin+ DC in Lamina Propria	0.16	FEV_1_(% pred)	-0.350	**0.018**
		Age	-0.076	0.525
		Gender	0.160	0.179
		Packyear	0.111	0.381
		Smoking status	0.078	0.503
		Inhaled CS	-0.093	0.542
		Oral CS	0.125	0.336

Langerin+ DC in Adventitia	0.40	FEV_1_(% pred)	-0.434	**0.001**
		Age	0.045	0.673
		Gender	0.217	**0.039**
		Packyear	-0.028	0.799
		Smoking status	0.115	0.271
		Inhaled CS	0.187	0.176
		Oral CS	0.072	0.542

Abbreviations: DC: dendritic cell; FEV1(% pred): forced expiratory volume in one second (% predicted); CS: corticosteroids.

β:standardized coefficient. Smoking status: current versus non-current smoker

Significant p values are marked in bold.

#### BDCA-1 positive DC

Representative cryosections are shown in figure 5 G-I. BDCA-1+ DC were mainly found in the epithelium and the lamina propria. The number of BDCA-1+ DC was significantly lower in the total airway wall of all COPD groups compared to never smokers (p = 0.042 for COPD GOLD I&II current smokers, p = 0.032 for COPD GOLD I&II ex-smokers and p < 0.001 for COPD GOLD III&IV ex-smokers) (figure 6C). Importantly, the number of these DC in the total airway wall was lower in COPD GOLD stage III/IV compared to the milder stages of the disease (p = 0.003 versus COPD GOLD I&II current smokers and p = 0.058 vs COPD GOLD I-II ex-smokers).

There were no significant differences between current and ex-smoking COPD GOLD I&II and between current and ex-smokers without airway obstruction. When focusing on the lamina propria and adventitia (fig 7H-I), the number of BDCA-1 positive DC was significantly lower in current smokers without airway obstruction compared to never smokers (p = 0.044 and p = 0.032, respectively) There was a significant positive correlation between the FEV1 (% predicted) and the number of BDCA-1+ DC in the epithelium (r_s_ 0.39; p < 0.001), lamina propria (r_s_ 0.35; p = 0.001), adventitia (r_s_ 0.36 ; p = 0.001) and total airway wall (r_s_ 0.40; p < 0.001). The association between FEV1 (% pred) and the number of BDCA-1 positive DC in the small airways was investigated by linear regression analysis (Table 5). This revealed that the decrease in BDCA-1 positive DC in the lamina propria is predominantly associated with the use of inhaled corticosteroids, which are mostly prescribed in the COPD patients with the lowest FEV1. In contrast, an independent association of FEV1 and BDCA-1+ DC was still observed in the epithelium and the adventitia. Gender did not influence the association between FEV1 and the number of BDCA-1 positive DC (data not shown).

Importantly, there was a trend towards an inverse correlation between the number of langerin+ DC in the total airway wall and the number of their known BDCA-1+ precursors: (r_s_ -0.21, p = 0.056).

**Table 5:** Linear Regression Analysis (Quantification BDCA-1+ DC in small airways)

DC surface marker	**R**^**2**^	Predictor Variable	β	p
BDCA-1+ DC in Total Airway Wall	0.08	FEV_1_(% pred)	-0.172	0.468
		Inhaled CS	0.045	0.130
		Oral CS	-0.172	0.826

BDCA-1+ DC in Epithelium	0.12	FEV_1_(% pred)	0.333	**0.034**
		Inhaled CS	0.064	0.710
		Oral CS	-0.110	0.420

BDCA-1+ DC in Lamina Propria	0.23	FEV_1_(% pred)	0.003	0.981
		Inhaled CS	-0.520	**0.002**
		Oral CS	0.100	0.418

BDCA-1+ DC in Adventitia	0.12	FEV_1_(% pred)	0.404	**0.011**
		Inhaled CS	0.062	0.724
		Oral CS	0.043	0.747

Abbreviations: DC: dendritic cell; FEV1(% pred): forced expiratory volume in one second (% predicted); CS: corticosteroids.

β:standardized coefficient.

Significant p values are marked in bold.

#### CD1a positive DC

Representative paraffin embedded sections showing CD1a+ DC are presented in figure 5J-L. CD1a + DC were mainly present in the adventitia and the lamina propria of the small airways. There were no significant changes in the number of CD1a+ DC in either the total airway wall (fig 6D), the epithelium, the lamina propria or the adventitia between the different groups (figure 7:J-K). No significant correlations with the post-bronchodilator FEV1 (% predicted) were found (data not shown).

### Factors involved in Langerhans-type DC differentiation and survival

The characteristics of the study population are shown in Table 6.

**Figure 8:**
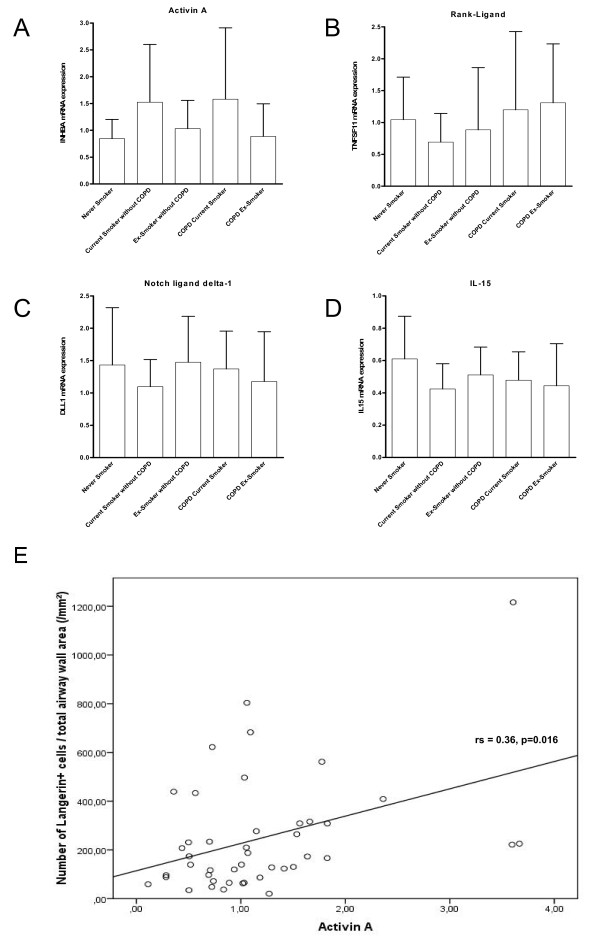
**mRNA expression in total lung of factors capable of inducing differentiation of monocytes towards Langerhans cells in never smokers, current smokers without COPD, ex-smokers without COPD, current smoking COPD patients and ex-smoking COPD patients**. (A) expression of Activin-A, (B) RANK-ligand, (C) notch-ligand delta-1 and (D) interleukin 15. mRNA expression is shown as the ratio of the number of transcripts of the gene of interest to the number of transcripts for the housekeeping genes. (E) Correlation of the mRNA expression levels of Activin-A, with the number of langerin positive cells in the total airway wall of the small airway. Correlation coefficient and p value are shown.

**Figure 9:**
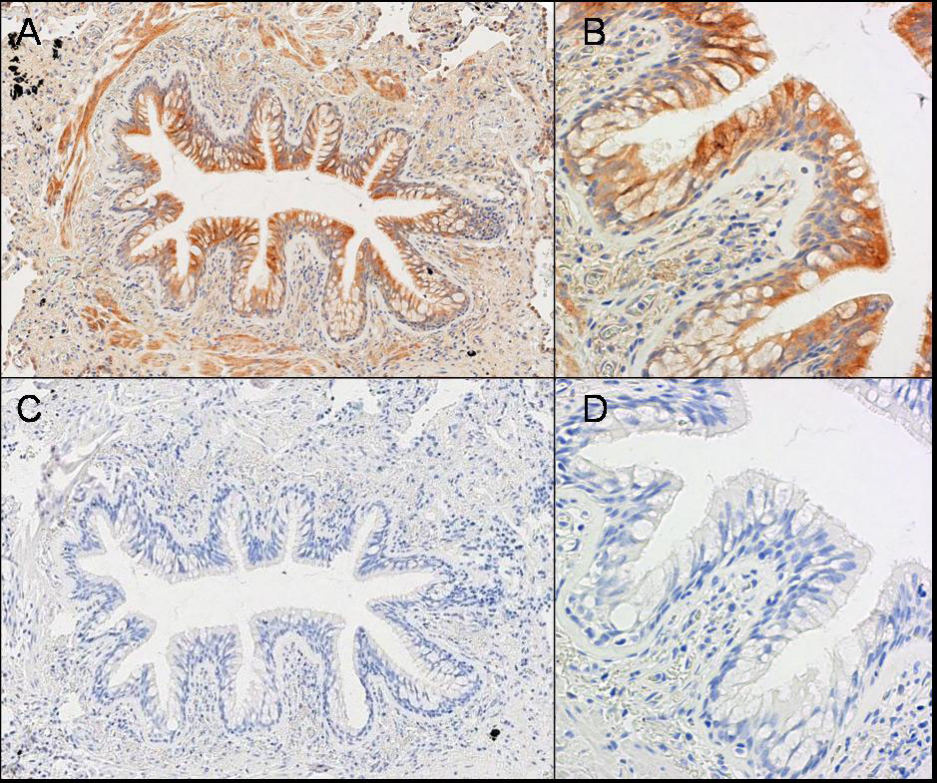
**Immunohistochemical staining for Activin-A in small airway of human lung (A)**. Detail of the airway epithelium (B). Isotype control staining is presented in (C) and (D). Activin-A is strongly expressed in the airway epithelium and smooth muscle layer. The mononuclear cell infiltrate also expresses Activin-A. A mild expression is also observed the connective tissue of the lamina propria and adventitia.

The results of the mRNA expression of the different factors (activin-A, RANK-Ligand, notch ligand delta-1 and IL-15) involved in LDC differentiation and survival are shown in figure 8A-D. For all the investigated factors, no significant differences in mRNA expression between groups were observed. However, there was a trend towards higher expression of activin-A in current smokers with COPD compared to never smokers and ex-smokers without COPD (p = 0.07 and p = 0.08 respectively). Moreover, there was a significant positive correlation between the expression of activin-A and the number of LDCs in the small airway (epithelium rs 0.33; p = 0.028, lamina propria rs 0.46 p = 0.002, adventitia rs 0.49; p = 0.001 and total airway wall rs 0.36 p = 0.016) (figure 8E). There were no significant correlations between the numbers of LDC and the expression of RANK-ligand, Notch ligand delta-1 or IL-15. Immunohistochemical staining of Activin-A confirms expression of this factor in the small airway at protein level, and shows its localization in the epithelium, the smooth muscle layer and in the mononuclear cellular infiltrate (figure 9).

**Table 6:** Characteristics of the Study Population (mRNA expression study) (n = 55)

	Never Smokers	Current Smokers without COPD	Ex-Smokers without COPD	COPD Current Smokers	COPD Ex-Smokers
**n**	10	7	10	17	11
**Sex ratio (M/F)**	3/7	3/4	8/2	14/3	11/0
**Age (years)**	59.5 (11.2)	53.1 (13.4)	63.6 (7.7)	63.9 (9.5)	69.3 (7.7)
**Smoking history (packyear)**	0	34.8 (12) *	37.5 (20.6) *	41.2 (16.9) †	56.1 (24.1) *
**Years quit smoking**	N.A.	0.0	9.0 (10.7) †	0.0 ‡	5.8 (6.0)†Δ
**FEV1 (%pred)**	97.3 (13)	101.8 (16.7)	112.2 (10.1)	68.3 (13.6)*†‡	68.8 (14.6)*†‡
**FEV1 (L)**	2.8 (0.7)	2.9 (0.4)	3.4 (0.7)	2.3 (0.6)‡	2.1 (0.5)*†‡
**FEV1/FVC (%)**	77.2 (8.1)	77.2 (4.9)	77.2 (6.1)	56.1 (7.0)*†‡	55.6 (9.2)*†‡
**LABA (Y/N)**	0/10	0/7	0/10	6/11	5/6
**Inhaled corticosteroids (Y/N)**	0	0	0	6/11	5/6
**Inhaled corticosteroid dose (μg BDP/24 h)**	0	0	0	538.5 (877.1)*†‡	663.6 (897.0)*†‡
**Systemic corticosteroids (Y/N)**	0/10	0/7	0/10	3/14	1/10
**Systemic corticosteroid dose (mg prednisolon/24 h)**	0	0	0	3.1 (10.0)	0.9 (3.0)

Data are expressed as mean (standard deviation);

* p < 0.05 vs never smoker; † p < 0.05 versus current smoker without COPD; ‡ p < 0.05 versus ex-smoker without COPD; Δ p < 0.05 versus COPD current smoker.

FEV1: forced expiratory volume in one second;

FVC: forced vital capacity;

LABA : long acting β 2 agonist; BDP: beclomethasone diproprionate.

## Discussion

This is the first study characterizing langerin+ Langerhans-type and DC-SIGN+ interstitial type DC as two separate populations in single cell suspensions of digested human lung tissue. Moreover, the extensive immunohistochemical study showed a selective accumulation of the LDC subset in small airways of current smokers and COPD patients, which correlated with its differentiation factor Activin-A. These data suggest a role for the LDC subset in the initiation of airway inflammation in susceptible smokers and perpetuation of this destructive process in COPD, even after smoking cessation.

Flowcytometric characterization of the segregation of LDC and intDC was confirmed in small airways using immunohistochemical double staining, which also showed that LDC are mainly present in the epithelium, whereas intDC are mainly localized in the lamina propria and adventitia. These findings are parallel to the known distribution of these two DC subsets in human skin [11].

**Figure 10:**
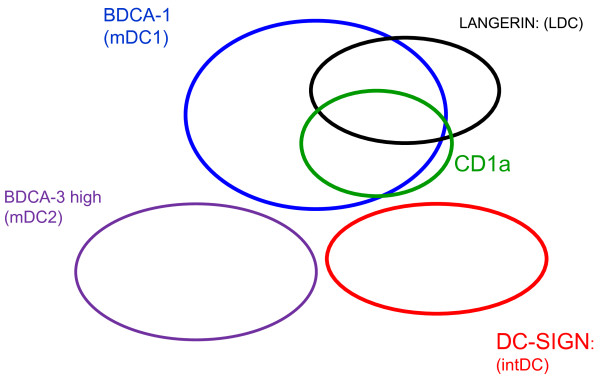
**Proposed schematic representation of the interrelationship of pulmonary myeloid dendritic cell subsets assessed by flowcytometry**. Pulmonary langerin positive Langerhans-type dendritic cells (LDC) are partially express CD1a and are closely related to BDCA-1 positive dendritic cells. These cells are separated from the interstitial type dendritic cells (intDC), which are identified by DC-SIGN and the myeloid dendritic cell type 2 (mDC2), identified by BDCA-3 high expression. Dimensions of the Venn-diagrams are not proportional to the absolute size of the different pulmonary DC subsets.

A schematic overview of the interrelationship between different pulmonary myeloid DC markers is provided in figure 10. Pulmonary LDC are closely related to the previously described pulmonary myeloid DC type 1 (mDC1) defined by BDCA-1. Indeed, in vitro studies on monocyte derived DC have shown that BDCA-1 and CD1a double positive DCs are direct precursors of these LDC [20]. CD1a, present on a subset of pulmonary BDCA-1+ DCs, is also present on a subgroup of LDC, but not all LDC co-express CD1a, suggesting that CD1a is not a good surrogate marker for pulmonary LDC. Moreover, this finding is supported by the distribution of CD1a in the small airways of human lungs which is completely different from langerin and BDCA-1 as CD1a positive cells are mainly present in the lamina propria and the adventitia. This difference in distribution of BDCA-1 and CD1a + DCs was also observed by Masten et al, showing higher quantification of CD1a positive DCs in the subepithelial regions compared to the epithelium [14].

In contrast, pulmonary intDC do not express BDCA-1 and CD1a, indicating that these DC are in a separate differentiation axis, more closely related to monocytes and macrophages, as hypothesized previously [12].

Although BDCA-3 can be expressed at an intermediate level on both the mDC1 related LDC and on intDC, BDCA-3 high expression is confined to a separate population of DC, that does not express BDCA-1, langerin, DC-SIGN or the pDC marker BDCA-2. This separate BDCA-3 high DC population could be regarded as a more accurate definition of the previously described myeloid DC type 2 (mDC2) [13]. It is unclear how this pulmonary mDC2 population is exactly related to the other DC subsets in terms of differentiation pathways, as there are currently no in vitro data available on this issue. Since we demonstrated that mDC2 also partially express CD14, we suggest that mDC2 are a fourth modality of differentiating monocytes (apart from macrophages, interstitial type DC and the mDC1-LDC axis).

A recent study by Tsoumakidou et al addressed the issue of different myeloid DC subsets in human lung digests by immunocytochemical staining of in vitro adhered pulmonary cells [34]. The target cell population of that study was mainly a mature CD83+ CD1a+ population. They also found that langerin, BDCA-1 and CD1a are not necessarily co-expressed. In addition, a substantial percentage of these cells also expressed BDCA-3. These results are generally in line with our findings. Unexpectedly, a high percentage of the cells in the study by Tsoumakidou et al also expressed the plasmacytoid DC (pDC) marker BDCA-2. This finding is in sharp contrast to the results of other research groups, including ours, showing that pDC are truly separated from myeloid DC. Our study has the advantage of the real-time simultaneous analysis of the different DC surface markers by flowcytometry, which generates more accurate results than the quantification of sequential immunocytochemical single stainings of adhered DC that could be phenotypically altered by the incubation in vitro.

Importantly, the relative proportions of different DC subsets differ, depending on the technique used for quantification. As lung digests contain cells from the different compartments of the lung specimen (airways, alveoli, lymphoid follicles, blood vessels and blood), the proportion of DC markers which are both expressed on circulating blood DC and tissue resident DC (such as BDCA-1) is generally higher compared to the tissue resident DC markers langerin and DC-SIGN in flowcytometric experiments. In contrast, when the focus is strictly on the small airway (in the immunohistochemical analysis), the tissue resident DC markers outnumber the BDCA-1 positive DC.

The immunohistochemical study of the different myeloid DC subsets in the small airways revealed a shift of the myeloid DC population towards a Langerhans phenotype with higher numbers of LDC in COPD patients compared to never smokers and ex-smokers without COPD. Moreover, the number of LDC further increased with the severity of the disease, confirming the results of our previous observations [22]. In accordance with previous studies in BAL [27,28], the number of LDC was higher in current smokers without COPD compared to never smokers or ex-smokers. In contrast, the number of LDC was not different between current smoking and ex-smoking COPD patients, supporting the concept of ongoing inflammation in COPD, despite smoking cessation.

Quantification of BDCA-1 positive DC revealed a completely different result with a significantly lower number of these DC in COPD patients compared to never smokers, especially in the lamina propria. Moreover, the number of BDCA-1+ DC further decreased with the severity of the disease. In line with the results of Soler et al [26], we found no differences in the number of epithelial BDCA-1+ cells between current smokers and ex-or never smokers. In addition, there was no effect of current smoking on the numbers of BDCA-1 positive DC in COPD patients. This marked divergence between the numbers of LDC and BDCA-1 positive DC could be explained by an alteration in the DC differentiation process. Indeed, adding TNF-alpha - a factor that is capable of driving the differentiation towards LDC - to a monocyte derived DC culture system, induces a lower expression of BDCA-1, compared to IL-4 generated DCs [35]. The fact that in our study the number of LDC tended to be inversely correlated with the number of BDCA-1+ DC in the small airways further supports this concept of an enhanced DC differentiation process towards a LDC phenotype in patients with COPD. Finally, this concept of enhanced LDC differentiation is compatible with the flowcytometric results on lung digests as shown in table 1. This revealed that the percentage of LDC expressing BDCA-1 tended to be lower in patients with moderate COPD (subject 3 and 6) compared to never smokers, suggesting that in COPD, BDCA-1 expression on LDC is reduced.

Quantification of CD1a, a marker present on both the BDCA-1+ precursors of LDC and on a subset of LDC, showed no differences between groups, indicating that smoking or COPD does not alter the number of CD1a positive DC in the small airways. These data confirm and extend the data of Soler et al in bronchiolar epithelium of smokers and non-smokers [26], and the previously published studies investigating the number of CD1a positive DC in bronchial biopsies and small airways of COPD patients [24,25]. These findings are in contrast with the clear accumulation of CD1a positive DCs in airways of asthmatics and of patients with diffuse panbronchiolitis [36,37].

To our knowledge, this is the first study evaluating the number of IntDC in small airways of smokers and COPD patients. Importantly, our study showed no significant differences in numbers of intDC in small airways between groups.

Taken together, these data suggest an alteration of the differentiation process of myeloid DC in small airways of COPD patients resulting in a selective accumulation of the LDC subset. The predominant accumulation of LDC in COPD patients is an important finding, as LDC are known to be potent activators of T helper1 cells and cytotoxic T cell responses, both involved in COPD pathogenesis [38]. Freeman et al recently showed that the production of CD8+ T cell attracting chemokines CCL-3 and CXCL-9 by CD1a positive lung DC (which are closely related to LDC) increases with COPD severity [39]. Moreover, recent publications also highlight the importance of LDC cells in activating Th17 cells [40], a T cell subset that could contribute to the adaptive immune response in COPD. In addition, LDC have different Toll-Like receptor expression compared to non- LDC, making them less capable to pick up certain microbial danger signals [41-43]. This could impair antimicrobial defence, leading to low grade infections that contribute to the destructive inflammation in COPD. This functional evidence on LDC is derived from skin DC or in vitro generated LDC. Further investigations to elucidate the functional differences between pulmonary Langerhans-type and non-Langerhans-type DC are warranted in order to better understand the role of the different DC subsets in the initiation and perpetuation of inflammation in COPD.

**Figure 11:**
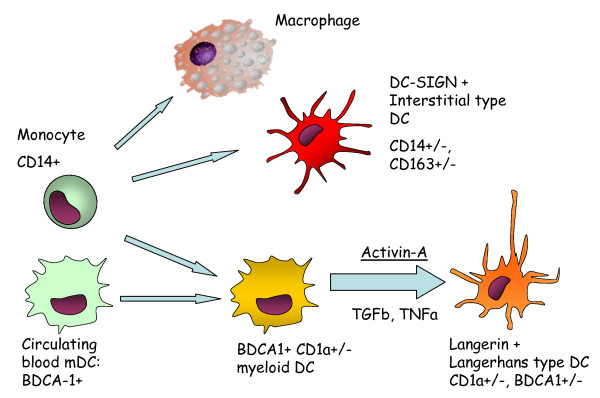
**Proposed mechanism of Dendritic Cell (DC) differentiation towards tissue resident DC in COPD**. Langerhans-type DC originate from their Blood Dendritic Cell Antigen 1 (BDCA-1)+ precursors under the influence of Transforming Growth Factor beta (TGFβ), Tumor Necrosis Factor Alpha (TNFα) and Activin-A. During this differentiation process, DC downregulate expression of BDCA-1. Enhanced differentiation towards Langerhans-type DC is present in COPD, whereas the differentiation axis towards DC-SIGN positive interstitial type DC is not altered in COPD.

Multiple factors such as chemokines and cytokines expressed by the airway epithelium, stromal cells and inflammatory cells under the influence of cigarette smoke and microbial stimuli can modulate the influx of DC precursors, their differentiation towards a certain phenotype and their capability to mature. It is also possible that in vivo, cigarette smoke could directly modulate certain DC functions. Previously, we reported an activation of the CCL-20/CCR6 axis in COPD which contributes to the influx of CCR6 expressing myeloid DC [22]. Monocytes and circulating myeloid blood DC are known precursors of LDC [19]. In vitro, the combination of Transforming Growth Factor beta (TGFβ) and Tumor Necrosis Factor alpha (TNFα) is crucial to induce a langerin expression in monocyte derived DC [18]. TGFβ and TNFα, both increased in the lungs of COPD patients [44,45], could therefore contribute to the enhanced differentiation towards LDC (figure 11).

Recently, other factors capable of inducing LDC differentiation and survival such as activin-A, notch ligand delta-1, RANK-ligand and IL-15 were identified in vitro and in the human skin. This study identifies pulmonary expressed activin-A as the differentiation factor correlating with the accumulation of LDCs in the small airways, highlighting its importance especially in current smokers. In addition, we describe for the first time the expression of activin-A in the small airways of human lungs, especially in the area that contains the highest concentration of langerin positive cells: the airway epithelium. Activin-A is a TGFβ superfamily member known for its activity on growth and differentiation of various cell types during organogenesis, and for its role in wound healing and inflammation [29]. Apart from inducing DC differentiation, activin-A is known to attenuate the pro-inflammatory response of DC in the context of stimulation with CD40 ligand, but not in the context of Toll-Like Receptor 4 stimulation [46]. Moreover, activin-A is also involved in promoting the chemotaxis of immature myeloid DC [47].

There are several factors that strengthen this study. First of all, this study is, to our knowledge, the largest study (85 patients) addressing the quantification of myeloid DC in human lungs, using different markers and taking into account not only the disease-effect (COPD), but also the current smoking effect. Secondly, as COPD is defined as a small airway disease, assessing the number of DC in this location yields more relevant results compared to endobronchial biopsies, which only sample the central airways. Thirdly, the interrelationship of the different myeloid DC markers was elucidated by flowcytometry and crucial segregations were confirmed on immunhistochemical staining. Finally, this study provides insight in new mechanisms that contribute to the alterations in the composition of the DC population in the small airways.

However, there are also several limitations to this study which should be addressed.

Firstly, tissue was obtained from surgical resection specimens from patients undergoing thoracotomy for solitary pulmonary lesions. In theory, the presence of this pathologic lesion could influence the number of DCs in the small airways. However, samples were obtained at maximum possible distance of this lesion, assuring the absence of retro-obstructive pneumonia or tumour invasion. Moreover, all groups, except GOLD stage III&IV contained patients with these lesions, minimizing their influence.

Secondly, the difference in gender ratio between the study groups (with a higher proportion of women in the never smoking group and a predominant male COPD population) could influence the results of this study. Recent publications suggest indeed a role for sex and gender in the susceptibility and pathogenesis of COPD [48]. However, significant associations between DC subsets and FEV_1 _were not influenced by adjusting for gender in the linear regression model, suggesting that our findings regarding LDC and BDCA-1 DC are not due to gender differences between groups.

Thirdly, the use of inhaled and systemic corticosteroids, especially in the most severe stages of COPD, could influence the DC numbers and differentiation. Indeed, several studies reported a decrease of circulating myeloid BDCA-1+ DC in patients treated with systemic corticosteroids [49,50]. Moreover, inhaled corticosteroids reduced the number of CD1a positive DCs in bronchial biopsies of atopic asthma patients [36]. In contrast, use of inhaled corticosteroids did not affect the number of CD1a positive DCs in bronchial biopsies of COPD patients [21]. Importantly, we demonstrated by linear regression analysis that the number of LDC in the small airways remained significantly associated with FEV_1_, even after adjustment for possible confounders such as the treatment with inhaled and oral corticosteroids. In contrast, we found that FEV_1 _was no longer associated with the number of BDCA-1 positive DC in lamina propria of the small airways when the treatment with corticosteroids was taken into account, suggesting that the lower number of BDCA-1 positive DC observed in COPD patients could at least be partially attributed to the treatment with corticosteroids, as these medications are mainly prescribed in the more severe stages of COPD.

Finally, identification of DC using immunohistochemical staining for a single marker is hazardous as some markers are also expressed on other cell types. For instance, BDCA-1 is also expressed on B cells, whereas DC-SIGN expression is also reported on macrophages. Therefore, only cells with a morphology compatible with a DC were regarded as positive cells.

## Conclusions

This flowcytometric and immunohistochemical study characterized for the first time pulmonary Langerhans-type DC, which are related to their known BDCA-1+ precursors and which are segregated from interstitial-type DC. In addition, we showed that DC differentiation is altered in small airways of current smokers and COPD patients with a selective accumulation of the Langerhans-type DC, correlating with the expression of Langerhans-type DC-inducing differentiation factor activin-A. This study identified the Langerhans-type DC subset as an interesting focus for future research in COPD pathogenesis.

## Competing interests

The authors declare that they have no competing interests.

## Authors' contributions

GRVP carried out the design and coordination of the study, performed flowcytometric analyses, carried out the immunohistochemical stainings for langerin, CD1a and DC-SIGN, quantified all immunohistochemical stainings, participated in the RNA extraction and RT-PCR, performed all statistical analysis, interpreted the data and drafted the manuscript. KRB participated in the RNA extraction, carried out the RT-PCR and helped to draft the manuscript. IKD participated in the design and coordination of the study, participated in the immunohistochemical stainings, helped to interpret the data and critically revised the manuscript. KDR participated in the quantification of the immunohistochemical staining and critically revised the manuscript. SMR participated in the immunohistochemical staining (BDCA-1) and critically revised the manuscript. CMVD critically revised the paper. GMV participated in the coordination of the study and critically revised the paper. FEV participated in the coordination of the study and critically revised the paper. GFJ participated in the coordination of the study, helped to interpret the data and critically revised the paper. GGB conceived the design of the study, participated in the coordination of the study, helped to interpret the data and helped to draft the manuscript. All authors read and approved the final manuscript.

## References

[B1] Rabe KF Hurd S Anzueto A Barnes PJ Buist SA Calverley P Global Strategy for the Diagnosis, Management, and Prevention of Chronic Obstructive Pulmonary Disease: GOLD Executive Summary Am J Respir Crit Care Med 2007 176 6 532 555 10.1164/rccm.200703-456SO 17507545

[B2] Mathers CD Loncar D Projections of global mortality and burden of disease from 2002 to 2030 PLoS Medicine 2006 3 11 e442 10.1371/journal.pmed.0030442 17132052 PMC1664601

[B3] Halpin DMG Miravitlles M Chronic Obstructive Pulmonary Disease: The Disease and Its Burden to Society Proc Am Thorac Soc 2006 3 7 619 623 10.1513/pats.200603-093SS 16963544

[B4] Di Stefano A Capelli A Lusuardi M Balbo P Vecchio C Maestrelli P Severity of airflow limitation is associated with severity of airway inflammation in smokers Am J Respir Crit Care Med 1998 158 4 1277 1285 9769292 10.1164/ajrccm.158.4.9802078

[B5] Lapperre TS Willems LN Timens W Rabe KF Hiemstra PS Postma DS Small airways dysfunction and neutrophilic inflammation in bronchial biopsies and BAL in COPD Chest 2007 131 1 53 59 10.1378/chest.06-0796 17218556

[B6] Barnes PJ Chronic obstructive pulmonary disease N Engl J Med 2000 343 4 269 280 10.1056/NEJM200007273430407 10911010

[B7] Saetta M Di Stefano A Turato G Facchini F Corbino L Mapp C CD8+ T-Lymphocytes in Peripheral Airways of Smokers with Chronic Obstructive Pulmonary Disease Am J Respir Crit Care Med 1998 157 3 822 826 9517597 10.1164/ajrccm.157.3.9709027

[B8] Gosman MM Willemse BW Jansen DF Lapperre TS van SA Hiemstra PS Increased number of B-cells in bronchialbiopsies in COPD Eur Respir J 2006 27 1 60 64 10.1183/09031936.06.00007005 16387936

[B9] Hogg JC Chu F Utokaparch S Woods R Elliott WM Buzatu L The nature of small-airway obstruction in chronic obstructive pulmonary disease N Engl J Med 2004 350 26 2645 2653 10.1056/NEJMoa032158 15215480

[B10] Strate BW van der Postma DS Brandsma CA Melgert BN Luinge MA Geerlings M Cigarette smoke-inducedemphysema: A role for the B cell? Am J Respir Crit Care Med 2006 173 7 751 758 10.1164/rccm.200504-594OC 16399994

[B11] Steinman RM Banchereau J Taking dendritic cells intomedicine Nature 2007 449 7161 419 426 10.1038/nature06175 17898760

[B12] Vermaelen K Pauwels R Pulmonary dendritic cells Am J Respir Crit Care Med 2005 172 5 530 551 10.1164/rccm.200410-1384SO 15879415

[B13] Demedts IK Brusselle GG Vermaelen KY Pauwels RA Identification and characterization of human pulmonary dendritic cells Am J Respir Cell Mol Biol 2005 32 3 177 184 10.1165/rcmb.2004-0279OC 15576669

[B14] Masten BJ Olson GK Tarleton CA Rund C Schuyler M Mehran R Characterization of myeloid and plasmacytoid dendritic cells in human lung J Immunol 2006 177 11 7784 7793 17114449 10.4049/jimmunol.177.11.7784

[B15] Tsoumakidou M Tzanakis N Papadaki HA Koutala H Siafakas NM Isolation of myeloid and plasmacytoid dendritic cells from human bronchoalveolar lavage fluid Immunol Cell Biol 2006 84 3 267 273 10.1111/j.1440-1711.2006.01428.x 16509829

[B16] Donnenberg VS Donnenberg AD Identification, rare-event detection and analysis of dendritic cell subsets in broncho-alveolar lavage fluid and peripheral blood by flow cytometry Front Biosci 2003 8 s1175 s1180 10.2741/1185 12957845

[B17] Ueno H Klechevsky E Morita R Aspord C Cao T Matsui T Dendritic cell subsets in health and disease Immunol Rev 2007 219 118 142 10.1111/j.1600-065X.2007.00551.x 17850486

[B18] Geissmann F Prost C Monnet JP Dy M Brousse N Hermine O Transforming growth factor beta1, in the presence of granulocyte/macrophage colony-stimulating factor and interleukin 4, induces differentiation of human peripheral blood monocytes into dendritic Langerhans cells J Exp Med 1998 187 6 961 966 10.1084/jem.187.6.961 9500798 PMC2212193

[B19] Ginhoux F Tacke F Angeli V Bogunovic M Loubeau M Dai XM Langerhans cells arise from monocytes in vivo Nat Immunol 2006 7 3 265 273 10.1038/ni1307 16444257 PMC4727824

[B20] Ito T Inaba M Inaba K Toki J Sogo S Iguchi T A CD1a+/CD11c+ subset of human blood dendritic cells is a direct precursor of Langerhans cells J Immunol 1999 163 3 1409 1419 10415041

[B21] D'hulst AI Vermaelen KY Brusselle GG Joos GF Pauwels RA Time course of cigarette smoke-induced pulmonary inflammation in mice Eur Respir J 2005 26 2 204 213 10.1183/09031936.05.00095204 16055867

[B22] Demedts IK Bracke KR Van Pottelberge GR Testelmans D Verleden GM Vermassen FE Accumulation of dendritic cells and increased CCL20 levels in the airways of patients with chronic obstructive pulmonary disease Am J Respir Crit Care Med 2007 175 10 998 1005 10.1164/rccm.200608-1113OC 17332482

[B23] Rogers AV Adelroth E Hattotuwa K Dewar A Jeffery PK Bronchial mucosal dendritic cells in smokers and ex-smokers with COPD: an electron microscopic study Thorax 2008 63 2 108 114 10.1136/thx.2007.078253 17875567

[B24] Verhoeven GT Hegmans JPJJ Mulder PGH Bogaard JM Hoogsteden HC Prins JB Effects of fluticasone propionate in COPD patients with bronchial hyperresponsiveness Thorax 2002 57 8 694 700 10.1136/thorax.57.8.694 12149529 PMC1746396

[B25] Tsoumakidou M Koutsopoulos AV Tzanakis N Dambaki K Tzortzaki E Zakynthinos S Decreased Small Airway and Alveolar CD83+ Dendritic Cells in COPD Chest 2009 19465512 10.1378/chest.08-2824

[B26] Soler P Moreau A Basset F Hance AJ Cigarette smoking-induced changes in the number and differentiated state of pulmonary dendritic cells/Langerhans cells Am Rev Respir Dis 1989 139 5 1112 1117 2712439 10.1164/ajrccm/139.5.1112

[B27] Casolaro MA Bernaudin JF Saltini C Ferrans VJ Crystal RG Accumulation of Langerhans' cells on the epithelial surface of the lower respiratory tract in normal subjects in association with cigarette smoking Am Rev Respir Dis 1988 137 2 406 411 3277501 10.1164/ajrccm/137.2.406

[B28] Bratke K Klug M Bier A Julius P Kuepper M Virchow JC Function-associated surface molecules on airway dendritic cells in cigarette smokers Am J Respir Cell Mol Biol 2008 38 6 655 660 10.1165/rcmb.2007-0400OC 18203971

[B29] Musso T Scutera S Vermi W Daniele R Fornaro M Castagnoli C Activin A Induces Langerhans Cell Differentiation In Vitro and in Human Skin Explants PLoS ONE 2008 3 9 e3271 10.1371/journal.pone.0003271 18813341 PMC2533393

[B30] Hoshino N Katayama N Shibasaki T Ohishi K Nishioka J Masuya M A novel role for Notch ligand Delta-1 as a regulator of human Langerhans cell development from blood monocytes J Leukoc Biol 2005 78 4 921 929 10.1189/jlb.1204746 16037408

[B31] Barbaroux JB Beleut M Brisken C Mueller CG Groves RW Epidermal receptor activator of NF-kappaB ligand controls Langerhans cells numbers and proliferation J Immunol 2008 181 2 1103 1108 18606662 10.4049/jimmunol.181.2.1103

[B32] Mohamadzadeh M Berard F Essert G Chalouni C Pulendran B Davoust J Interleukin 15 skews monocyte differentiation into dendritic cells with features of Langerhans cells J Exp Med 2001 194 7 1013 1020 10.1084/jem.194.7.1013 11581322 PMC2193478

[B33] Vandesompele J De PK Pattyn F Poppe B Van RN De PA Accurate normalization of real-time quantitative RT-PCR data by geometric averaging of multiple internal control genes Genome Biol 2002 3 7 RESEARCH0034 10.1186/gb-2002-3-7-research0034 12184808 PMC126239

[B34] Tsoumakidou M Kemp SJ Thorley AJ Zhu J Dewar A Jeffery PK Expression of blood dendritic cell antigens (BDCAs) by CD1a+ human pulmonary cells Respiratory Medicine 2009 103 6 935 938 10.1016/j.rmed.2009.02.006 19328670

[B35] Pickl WF Majdic O Kohl P Stockl J Riedl E Scheinecker C Molecular and functional characteristics of dendritic cells generated from highly purified CD14+ peripheral blood monocytes J Immunol 1996 157 9 3850 3859 8892615

[B36] Moller GM Overbeek SE van Helden-Meeuwsen CG Van Haarst JM Prens EP Mulder PG Increased numbers of dendritic cells in the bronchial mucosa of atopic asthmatic patients: downregulation by inhaled corticosteroids Clin Exp Allergy 1996 26 5 517 524 10.1111/j.1365-2222.1996.tb00571.x 8735863

[B37] Todate A Chida K Suda T Imokawa S Sato J Ide K Increased Numbers of Dendritic Cells in the Bronchiolar Tissues of Diffuse Panbronchiolitis Am J Respir Crit Care Med 2000 162 1 148 153 10903234 10.1164/ajrccm.162.1.9907015

[B38] Merad M Ginhoux F Collin M Origin, homeostasis and function of Langerhans cells and other langerin-expressing dendritic cells Nat Rev Immunol 2008 8 12 935 947 10.1038/nri2455 19029989

[B39] Freeman CM Curtis JL Chensue SW CC chemokine receptor 5 and CXC chemokine receptor 6 expression by lung CD8+ cells correlates with chronic obstructive pulmonary disease severity Am J Pathol 2007 171 3 767 776 10.2353/ajpath.2007.061177 17640964 PMC1959492

[B40] Mathers AR Janelsins BM Rubin JP Tkacheva OA Shufesky WJ Watkins SC Differential capability of human cutaneous dendritic cell subsets to initiate Th17 responses J Immunol 2009 182 2 921 933 19124735 10.4049/jimmunol.182.2.921

[B41] Rozis G Benlahrech A Duraisingham S Gotch F Patterson S Human Langerhans' cells and dermal-type dendritic cells generated from CD34 stem cells express different toll-like receptors and secrete different cytokines in response to toll-like receptor ligands Immunology 2008 124 3 329 338 10.1111/j.1365-2567.2007.02770.x 18194273 PMC2440827

[B42] Aar AMG van der Sylva-Steenland RMR Bos JD Kapsenberg ML de Jong EC Teunissen MBM Cutting Edge: Loss of TLR2, TLR4, and TLR5 on Langerhans Cells Abolishes Bacterial Recognition J Immunol 2007 178 4 1986 1990 17277101 10.4049/jimmunol.178.4.1986

[B43] Flacher V Bouschbacher M Verronese E Massacrier C Sisirak V Berthier-Vergnes O Human Langerhans cells express a specific TLR profile and differentially respond to viruses and Gram-positive bacteria J Immunol 2006 177 11 7959 7967 17114468 10.4049/jimmunol.177.11.7959

[B44] Takizawa H Tanaka M Takami K Ohtoshi T Ito K Satoh M Increased expression of transforming growth factor-beta1 in small airway epithelium from tobacco smokers and patients with chronic obstructive pulmonary disease (COPD) Am J Respir Crit Care Med 2001 163 6 1476 1483 11371421 10.1164/ajrccm.163.6.9908135

[B45] Keatings VM Collins PD Scott DM Barnes PJ Differences in interleukin-8 and tumor necrosis factor-alpha in induced sputum from patients with chronic obstructive pulmonary disease or asthma Am J Respir Crit Care Med 1996 153 2 530 534 8564092 10.1164/ajrccm.153.2.8564092

[B46] Robson NC Phillips DJ McAlpine T Shin A Svobodova S Toy T Activin-A: a novel dendritic cell-derived cytokine that potently attenuates CD40 ligand-specific cytokine and chemokine production Blood 2008 111 5 2733 2743 10.1182/blood-2007-03-080994 18156495

[B47] Salogni L Musso T Bosisio D Mirolo M Jala VR Haribabu B Activin A induces dendritic cell migration through the polarized release of CXC chemokine ligands 12 and 14 Blood 2009 113 23 5848 5856 10.1182/blood-2008-12-194597 19339694 PMC13127294

[B48] Han MK Postma D Mannino DM Giardino ND Buist S Curtis JL Gender and chronic obstructive pulmonary disease: why it matters Am J Respir Crit Care Med 2007 176 12 1179 1184 10.1164/rccm.200704-553CC 17673696 PMC2720110

[B49] Rozkova D Horvath R Bartunkova J Spisek R Glucocorticoids severely impair differentiation and antigen presenting function of dendritic cells despite upregulation of Toll-like receptors ClinicalImmunology 2006 120 3 260 271 10.1016/j.clim.2006.04.567 16765091

[B50] Bosma BM Metselaar HJ Tra WM Mancham S Kuipers EJ Tilanus HW Impairment of circulating myeloid dendritic cells in immunosuppressed liver transplant recipients Clin Exp Immunol 2007 149 3 525 534 17645770 10.1111/j.1365-2249.2007.03449.x PMC2219320

